# Fat Depots, Free Fatty Acids, and Dyslipidemia

**DOI:** 10.3390/nu5020498

**Published:** 2013-02-07

**Authors:** Jon O. Ebbert, Michael D. Jensen

**Affiliations:** 1 Division of Primary Care Internal Medicine, Mayo Clinic, 200 First Street SW, Rochester, MN 55905, USA; E-Mail: ebbert.jon@mayo.edu; 2 Division of Endocrinology, Mayo Clinic, 200 First Street SW, Rochester, MN 55905, USA

**Keywords:** free fatty acid, fat depots, dyslipidemia

## Abstract

Body fat deposition and excess free fatty acid (FFA) metabolism contribute to dyslipidemia and the adverse health consequences of obesity. Individuals with upper body obesity have impaired functioning of adipocytes, the primary fatty acid storage site. Excess visceral fat is strongly associated with impaired suppression of FFA release in response to insulin, as well as with hypertriglyceridemia and low concentrations of high density lipoprotein (HDL) cholesterol. High FFA concentrations can induce insulin resistance in muscle and liver. Furthermore, failure of hyperinsulinemia to normally suppress FFA is associated with impaired carbohydrate oxidation and muscle glucose storage, reduced hepatic insulin clearance and elevated triglycerides. Understanding the impact of body fat distribution on FFA metabolism and dyslipidemia is critical for determining the link between overweight and obesity and cardiovascular disease risk. In the current review, we will explore the relationship between adipose tissue, body fat depots, and FFA metabolism.

## 1. Introduction

Independent of total body fat, body fat distribution is an important and established risk factor for cardiovascular disease (CVD). Clinical measures of abdominal obesity, also referred to by some as upper body obesity, such as waist-to-hip ratio and waist circumference, are directly associated with progression of atherosclerosis [1] and the risk for acute coronary events [2]. For women and men, waist hip ratios of >0.85 and >0.95, respectively, identify populations at greater risk for insulin resistance/hyperinsulinemia, hypertension and dyslipidemia [3].

The majority of adults with abdominal obesity as defined by waist-hip ratio also have disproportionate amounts of omental and mesenteric (visceral) fat. Visceral fat is considered by many to behave as an ectopic fat depot, accumulating triglycerides (TG’s) when body fat storage needs exceed the capacity of subcutaneous fat depots to function normally [4]. Abnormally functioning subcutaneous fat exhibits reduced energy storage capacity combined with excess free fatty acid (FFA) release. These conditions, combined with increased infiltration of fatty tissue with inflammatory cells, release of cytokines, and reduced production of salutary adipokines, may account for increased CVD risk.

Visceral adiposity is strongly associated with the metabolic abnormalities that increase CVD risk. However, the explanation for this association is incompletely understood. One hypothesis is that visceral fat directly produces substances that cause the metabolic derangements associated with increased CVD risk in obesity [5,6,7]. Another hypothesis is that visceral fat is largely a marker for excess FFA release, largely by subcutaneous, but also by visceral, adipose tissue. In turn, the exposure of lean tissues to high FFA concentrations is thought to contribute to the metabolic abnormalities observed with increased visceral adiposity. In the postabsorptive state, fatty acids are the preferred fuel source for the heart [8], liver [9] and skeletal muscle [10], yielding large quantities of adenosine triphosphate (ATP). In the pathologically obese state, however, elevated fasting FFA and impaired FFA suppression are associated with hypertriglyceridemia [11,12,13,14].

One of the classic characteristics of obesity is dyslipidemia characterized by high levels of TG’s in very-low-density lipoproteins (VLDL) and low levels of high-density lipoprotein (HDL) cholesterol [15]. This type of dyslipidemia is a valuable predictor for the development of CVD, therefore, an understanding of the impact of body fat distribution on FFA metabolism and dyslipidemia is crucial for our understanding of how overweight and obesity increase the risk for CVD. In the current review, we will explore the relationship between adipose tissue, body fat depots, and FFA metabolism in the lean and obese states. We will also describe the relationship between FFA, body fat distribution and the dyslipidemia contributing to the CVD risk burden in obesity.

## 2. Adipose Tissue and Free Fatty Acids

Adipose tissue plays a dynamic and critical role in fuel metabolism. Adipocytes produce and secrete a broad array of proteins and other molecules such as leptin, adiponectin, tumor necrosis factor-α (TNF-α), and interleukin-1β (IL-1β) [16]. Fat storage and mobilization are highly coordinated activities occurring constantly in adipocytes, not just in response to over-eating. Chylomicron triglyceride-fatty acids derived from dietary fat that are not immediately oxidized are stored primarily in adipose tissue as TG. In turn, adipose tissue lipolysis supplies FFA as fuel to tissues and organs that can oxidize fatty acids. Adipocyte lipolysis, resulting in the liberation of FFA is potently regulated by insulin and catecholamines. Buffering the daily flux of circulating FFA occurs by suppressing the release of FFA into the circulation and by increasing TG clearance via increasing lipoprotein lipase activity [17]. Supply and storage of FFA occurs at different rates and amounts in obese and lean individuals depending upon the anatomical location of the adipose tissue, commonly referred to as “fat depots”.

## 3. Fat Depots

Fat depots can broadly be functionally and anatomically grouped into lower body fat, upper body subcutaneous fat, and visceral/intra-abdominal fat. In normal weight adults, upper body and lower body subcutaneous fat comprise the majority of total body fat [18]. Lower body fat is anatomically designated as adiposity below the inguinal ligament and the posterior superior iliac crest. Potential subdivisions of lower body include gluteal, subcutaneous thigh and calf fat, as well as adipose tissue found between muscles, also known as “marbling”. Although leg fat in general is not associated with increased cardiovascular risk, increased marbling is linked to metabolic abnormalities. In our opinion, marbling of muscle with adipose is another form of ectopic fat. Upper body subcutaneous fat includes deep and superficial truncal fat, arm and breast depots. The two anatomically distinct abdominal adipose tissue depots are the superficial and deep subcutaneous fat [19]; deep subcutaneous fat is correlated with visceral fat and more metabolic abnormalities. We examined whether deep and superficial abdominal subcutaneous fat differ with respect to meal fat storage and found them to behave quite similarly [20], suggesting to us that these subdivisions of abdominal subcutaneous fat may not be that functionally that different. Depots within the abdomen and chest that are correlated with metabolic abnormalities include omental and mesenteric fat (whose venous drainage is into the portal vein), perinephric and pericardial fat, which do not deliver blood to the portal vein [4]. Interest has developed regarding smaller depots, such as pericardial [21] fat, have direct metabolic effects on the heart or whether, like visceral and marbling fat, these are primarily ectopic markers of adipose dysfunction.

## 4. Obesity, Fat Depots, and FFA

Obesity is often associated with impaired functioning of adipose tissue. If lipolysis per kilogram of fat were the same in obese and non-obese individuals, the increase in plasma FFA concentrations would be much more striking in obesity. Fortunately, there appears to be some down-regulation of lipolytic rates per kilogram of adipose tissue such that in obesity plasma FFA are only 20%–30% greater than normal in the absence of type 2 diabetes [22,23,24,25]. However, in some circumstances expanded fat depots are actually less efficient in storing dietary fatty acids, which may expose lean tissues to excess chylomicron triglycerides and therefore to greater risk of ectopic fat accumulation. As mentioned, excess visceral fat is associated with greater metabolic risk [26,27] than is accumulation of fat in the lower body [28,29].

Excess FFA contributes to the adverse health consequences of obesity. It is not as simple as merely greater rates of whole body lipolysis leading to adverse metabolic consequences, however. For example, adipose tissue FFA release is significantly (~40%) greater in women than men relative to energy needs and fat oxidation [30], yet FFA concentrations are marginally higher and metabolic health is actually somewhat better in women. This may relate to the offsetting “buffering” properties of adipose tissue in women; direct adipose tissue FFA storage is substantially greater in women than men [26,31]. It is well established, however, that high levels of FFA can mediate insulin resistance in muscle [32,33] and liver [31,34]. Failure to suppress FFA is associated with inhibition of carbohydrate oxidation and glycogen synthesis in muscle during hyperinsulinemia [35], reduced clearance of insulin by the liver [36], and elevated VLDL-triglyceride production [37].

Compared with lean women, women with upper body obesity have lower basal adipose tissue lipolysis per kilogram of fat, but systemic FFA release is greater because of their higher fat mass [24]. FFA release per kilogram of fat is even further reduced in lower body obese women, such that systemic FFA release relative to fat free mass or resting energy expenditure is very similar to non-obese women. Upper body subcutaneous fat, being the primary source of FFA, is the primary culprit for excess FFA release in upper body obesity. Leg adipose tissue lipolysis and FFA storage in upper body subcutaneous fat depots is similar between upper body obese men and upper body obese women. 

Upper body obesity is associated with increased FFA release in both the postabsorptive and postprandial states, however, we believe the true hallmark of upper body/visceral obesity is the impaired suppression of FFA release in response to insulin [38] and meals [39,40] compared to non-obese or lower body obese adults. In upper body obesity and type 2 diabetes, the vast majority of excess FFA release during hyperinsulinemic conditions is from the upper body subcutaneous fat rather than visceral fat [39]. Postprandial FFA concentrations are 3-fold greater in upper body obese individuals [39,40] implying that upper body adipocytes are more resistant to the antilipolytic effects of insulin.

## 5. Fat Depots and Liver FFA Metabolism

**Figure 1:**
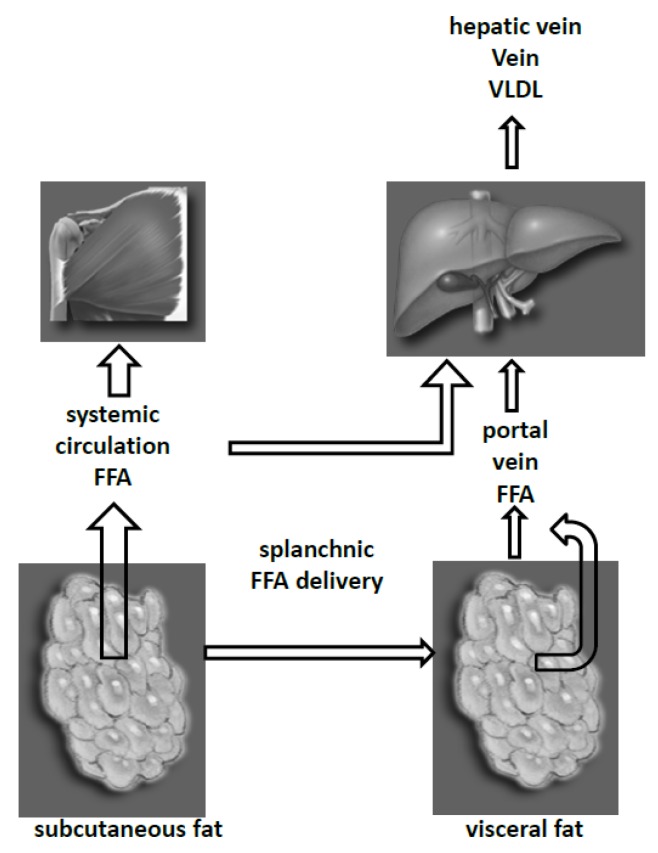
FFA Delivery from Body Fat Depots.

Expanded visceral fat depots and hyperinsulinemia do have adverse effects on the liver. The liver takes up a large proportion of FFA entering the splanchnic bed through the portal vein [41,42], with only a small fraction of FFA being taken up by non-hepatic splanchnic. We found that the proportion of FFA delivered to the liver coming from visceral adipose tissue lipolysis increases as a function of visceral fat mass, more so in women than men [32]. In some extreme viscerally obese women, up to 50% of postabsorptive hepatic FFA delivery is from visceral fat, although on average only approximately 20% of hepatic FFA delivery is from visceral fat [43]. Abnormally high levels of FFA presented to the liver stimulate the production of hepatic VLDL-TG (Figure 1) [9,37,44]. Thus, persons with greater amounts of visceral fat are likely to have selectively greater abnormalities in hepatic metabolism.

## 6. Fat Depots VLDL and LDL

The dyslipidemia of obesity is characterized by hypertriglyceridemia, an established independent risk factor for CVD [45]. Each 90 mg/dL increase in plasma triglyceride concentration is associated with a 32% increase in CVD risk in men (relative risk (RR) = 1.30; 95% confidence interval (CI): 1.25 to 1.35) and a 76% increase in women (RR = 1.69; 95% CI: 1.45 to 1.97) [46]. The association of hypertriglyceridemia is greater with visceral fat depots than subcutaneous fat depots [47], possibly because of the hepatic FFA delivery differences described above. Elevated waist circumference, a marker of expanded visceral fat depots, and hypertriglyceridemia have been described as “hypertriglyceridemic waist” [48].

One function of VLDL is to serve as a means for the liver to export excess energy in the form of triglycerides. VLDL assembly is, therefore, dependent upon the availability of TG [49]. Hepatic TG derives largely from uptake of circulating FFA, with the uptake of VLDL and chylomicron remnants, as well as de novo synthesis of fatty acids from sugars [49] providing lesser portions. The hypertriglyceridemia of obesity and obesity-related insulin resistance is primarily due to an overproduction of VLDL, although at some level VLDL clearance decreases as tissue lipoprotein lipase (LPL) becomes saturated. Under these more extreme circumstances of saturated LPL, postprandial accumulation chylomicrons and other TG-rich lipoprotein remnants occurs [50]. Hypertriglyceridemia is associated with TG enrichment of low-density lipoprotein (LDL) and HDL cholesterol [51]. TG enrichment is associated with low concentrations of high-density lipoprotein HDL cholesterol levels and higher concentrations of small, dense LDL particles. Dense LDL particles contain less cholesterol, less cholesterol is cleared through the LDL pathway, and a higher proportion of cholesterol is cleared through the VLDL pathway [52]. Clearance of VLDL may occur through the atherogenic routes such as macrophages and smooth muscle cells [52] which increase the risk for CVD. 

As noted above, is appears that omental and mesenteric fat, because of their portal vein venous drainage, play an important role in abnormalities of hepatic lipid metabolism. There is evidence that visceral fat depots are more resistant to insulin than leg fat and upper body subcutaneous fat [53], and that the combination of elevated insulin and FFA drive the production and secretion of VLDL from the liver. Although the majority of FFA delivery to the liver is from upper body subcutaneous fat, visceral fat can contribute up to 50% of hepatic FFA delivery in persons with large visceral depots [43]. Plasma FFA concentrations and hepatic FFA delivery are increased in insulin resistance [54,55]. Increased hepatic production of VLDL apoC-III is characteristic of subjects with higher fat mass and insulin resistance and is strongly correlated with plasma concentration and production level of VLDL TG [56].

## 7. Fat Depots and HDL Cholesterol

Dyslipidemia contributes substantially to the CVD risk associated with obesity. In obese adults, adipose depots contain over 50% of total body cholesterol [57]. Changes in body fat depots have a profound impact on lipid transport, plasma lipoprotein composition and concentrations [3]. Obesity adversely effects serum HDL cholesterol concentrations. The correlation of low HDL cholesterol with obesity is strongest with expanded visceral fat depots [58]. Epidemiologic studies have clearly demonstrated that those with higher plasma HDL cholesterol concentrations have lower CVD risk [59,60]. Each 5 mg/dL decrement in HDL cholesterol is associated with a 14% greater risk of CVD [61]. The converse is true as well, such that CVD risk decreases by 3% for every 1 mg/dL increase in HDL cholesterol [62]. Reverse cholesterol transport (RCT) has been proposed as the central anti-atherogenic effect of HDL particles reducing CVD risk [63], although some skepticism has recently been expressed that this is the sole reason for this association. 

Lipoprotein metabolism is partially regulated by the triglyceride lipases lipoprotein lipase (LPL) and hepatic triglyceride lipase (HTGL). High post-heparin LPL activity has been associated with high plasma concentrations of HDL cholesterol [64,65,66] and high post-heparin HTGL has been associated with low serum concentrations of HDL cholesterol [67,68]. Total body fat is associated with lower levels of LPL activity [69]. Independent of total body fat, an expanded visceral fat depot is correlated with high HTGL activity [70]. This effect may be mediated through increased androgen exposure causing simultaneous increases in visceral adiposity and HTGL activity [3]. Total body obesity combined with an expanded visceral fat depot results in significant reductions in HDL cholesterol resulting in an increased risk for CVD.

Expanded fat depots may be adversely effecting serum concentrations of HDL cholesterol by influencing HDL metabolism. The overproduction of FFA and VLDL-TG reduces plasma HDL-cholesterol concentrations [50]. Elevated plasma concentrations of VLDL appear to drive the transfer of TG to HDL. Obesity increases hepatic lipase (HL) [71] which hydrolyzes TG-rich HDL and releases lipid-poor apoA-I leading to the formation of remnant HDL particles [72]. These remnants are likely to be cleared by the kidney. 

Obesity and pathologically increased fat depots adversely affect the functionality and effectiveness of HDL particles. Expanded visceral fat depots, for example, decrease the anti-apoptotic activity of HDL particles [73].

## 8. Conclusions

Obesity, and specifically upper body/visceral obesity, is associated with insulin resistance, dyslipidemia and increased cardiovascular risk. Abnormal regulation of subcutaneous adipose tissue lipolysis in those with visceral obesity is likely to play an important role in these abnormalities, although dysregulation of adipokine secretion may also be important. Increased visceral fat, as well as being a good marker for subcutaneous adipose tissue insulin resistance, may also selectively affect hepatic metabolism via greater portal vein FFA delivery. In contrast, insulin resistance in skeletal muscle and abnormal insulin secretion is largely driven by abnormal upper body subcutaneous adipose tissue lipolysis. It will be interesting to determine if treatments that selectively improve the regulation of upper body subcutaneous fat or visceral fat may have differential benefits on lipids. 
